# Supplementary material for: An in silico investigation of menthol metabolism
Source: PLoS One. 2019 Sep 27;14(9):e0216577. doi: 10.1371/journal.pone.0216577 (PMC6764827; doi:10.1371/journal.pone.0216577)

Calculation procedure	Name	Quantity	Unit	Ref
1. Optimized in gas phase at B3LYP/6-311++G(d,p)	Hartree	627.5094742	kcal mol^-1/AU	2014 CODATA
2. Frequency calculation in gas phase at B3LYP/6-311++G(d,p)	Temp	298.15	K	Room Temp
3. Single point energy calculation in water (SMD) at B3LYP/6-311++G(d,p)	Faraday constant	96485.3329	C/mol	2014 CODATA
	calorie	4.184	J/cal	ISO 31-4:1992
Q-CHEM Developer Version 5.1	R	8.3144598	J mol^-1 K^-1	2014 CODATA
Program default output: STANDARD THERMODYNAMIC QUANTITIES AT 298.15 K AND 1.00 ATM	R	0.082057338	L atm mol^-1 K^-1	2014 CODATA
	[H2O]	55.34	M	Handbook of Chemistry and Physics, CRC press, 64th Ed
	∆G o->*	0.0030	AU	Limpanuparb2017 Section 2.4
	∆G H2O	0.0038	AU	Limpanuparb2017 Section 2.4

The energies were adjusted to solution standard state conditions * – 1M, 298.15 K – with the exception of water where 55.34 M was used.

			E+G/$C$1+C$9	F+I/$C$1-$C$2*J/1000/$C$1	Q-CHEM output at line: Final energy is	(9) = (6) - (0)	Zero point vibratio	Total Enthalpy:	Total Entropy:						
Formula/Filename	Number in the paper	Other name	G^*_{soln}	G_{gas}	E_{elec}	ΔG_{solv}	ZPE	H_{corr}	S	# Imag	Ref				Conclusion
			AU	AU	AU	kcal/mol	kcal/mol	kcal/mol	cal/mol/K		17	18	19	This paper	
C6H10O7			-761.357	-761.313	-761.452	-29.6	111.6	120.4	112.2	0					
H2			-1.175	-1.181	-1.180	1.6	6.3	8.4	31.1	0					
H2O			-76.462	-76.455	-76.459	-8.6	13.4	15.7	45.1	0					
H3O+			-76.867	-76.716	-76.731	-96.4	21.5	23.9	48.4	0					
O2			-150.324	-150.324	-150.309	-2.0	2.3	4.4	46.8	0					
O4S-2			-699.504	-699.129	-699.116	-237.4	9.0	12.3	68.8	0					
Oeeee	63		-1153.129	-1153.099	-1153.488	-20.8	276.5	291.7	159.2	0	X	X	F		Blue
oeeOe-R	70		-1228.369	-1228.332	-1228.725	-25.5	280.0	295.6	162.7	0	X	I	F		Blue
Oeeoe-R	68		-1228.370	-1228.334	-1228.726	-25.0	280.4	296.5	168.9	0	X	I	F		Blue
oeeOe-S	71		-1228.372	-1228.337	-1228.726	-24.2	279.8	296.1	173.6	0	X	I	F		Blue
Oeeoe-S	69		-1228.364	-1228.331	-1228.725	-22.3	280.4	296.3	165.2	0	X	I	F		Blue
oeeXe-R	88		-1302.455	-1302.408	-1302.775	-31.8	267.4	284.5	180.3	0	X	X	F		Blue
Oeexe-R	84		-1302.461	-1302.412	-1302.785	-32.7	268.1	284.3	168.4	0	X	X	F		Blue
oeeXe-S	89		-1302.451	-1302.406	-1302.777	-30.1	268.1	285.0	176.6	0	X	X	F		Blue
Oeexe-S	85		-1302.459	-1302.410	-1302.784	-32.6	268.5	284.6	167.9	0	X	X	F		Blue
Oeeye-R	80		-1227.187	-1227.154	-1227.520	-23.0	264.7	280.8	170.9	0	X	X	F		Blue
Oeeye-S	81		-1227.187	-1227.153	-1227.518	-23.2	264.5	280.6	171.4	0	X	X	F		Blue
Oeeyy	102		-1301.225	-1301.179	-1301.525	-31.0	252.6	269.2	173.7	0	X	X	F		Blue
oeOee	67		-1228.372	-1228.336	-1228.730	-24.6	279.4	294.9	159.9	0	X	I	F		Blue
Oeoee	66		-1228.383	-1228.344	-1228.734	-26.4	279.1	295.4	170.1	0	X	I	F		Blue
oOeee	65		-1228.375	-1228.331	-1228.720	-29.6	279.6	296.2	174.1	0	X	X	F		Blue
Ooeee	64		-1228.373	-1228.328	-1228.717	-30.0	279.4	296.1	175.1	0	X	X	F		Blue
ooeOe-R	76		-1303.618	-1303.563	-1303.957	-36.2	283.3	300.3	178.0	0	X	X	X	I	Black
oOeoe-R	74		-1303.620	-1303.568	-1303.961	-34.4	283.1	300.1	179.6	0	X	X	X	I	Black
Ooeoe-R	72		-1303.609	-1303.556	-1303.948	-35.1	282.6	300.0	180.4	0	X	X	X	I	Black
ooeOe-S	77		-1303.617	-1303.564	-1303.958	-34.6	282.9	300.0	177.5	0	X	X	X	I	Black
oOeoe-S	75		-1303.615	-1303.563	-1303.959	-34.3	283.7	300.6	176.2	0	X	X	X	I	Black
Ooeoe-S	73		-1303.602	-1303.549	-1303.944	-35.3	283.4	300.4	175.9	0	X	X	X	I	Black
ooeYe-5A-RR	94		-1302.415	-1302.364	-1302.736	-33.9	269.1	285.5	174.1	0	X	X	F		Blue
oOeye-5A-RR	90		-1302.426	-1302.377	-1302.750	-32.7	269.4	285.6	173.3	0	X	X	F		Blue
ooeYe-5A-RS	95		-1302.417	-1302.371	-1302.748	-30.2	270.0	285.9	165.9	0	X	X	F		Blue
oOeye-5A-RS	91		-1302.425	-1302.376	-1302.749	-32.8	269.5	285.7	173.4	0	X	X	F		Blue
ooeYe-5A-SR	96		-1302.428	-1302.379	-1302.751	-32.5	269.2	285.6	174.7	0	X	X	F		Blue
oOeye-5A-SR	92		-1302.427	-1302.378	-1302.752	-32.6	269.4	285.6	171.9	0	X	X	F		Blue
ooeYe-5A-SS	97		-1302.419	-1302.372	-1302.745	-31.9	269.2	285.5	171.9	0	X	X	F		Blue
oOeye-5A-SS	93		-1302.428	-1302.378	-1302.753	-33.1	269.3	285.0	165.4	0	X	X	F		Blue
oXeee-R	86		-1302.443	-1302.391	-1302.762	-34.4	268.2	285.0	175.3	0	X	X	F		Blue
Oxeee-R	82		-1302.455	-1302.401	-1302.772	-35.7	268.1	284.9	175.6	0	X	X	F		Blue
oXeee-S	87		-1302.441	-1302.390	-1302.759	-33.6	267.6	284.6	177.4	0	X	X	F		Blue
Oxeee-S	83		-1302.460	-1302.409	-1302.777	-33.6	267.8	284.7	181.0	0	X	X	F		Blue
Oyeee-R	78		-1227.182	-1227.140	-1227.504	-28.7	264.4	280.7	173.9	0	X	X	F		Blue
Oyeee-S	79		-1227.182	-1227.140	-1227.504	-28.4	264.0	280.4	174.7	0	X	X	F		Blue
Oyeye-RR	98		-1301.228	-1301.181	-1301.524	-31.7	252.1	269.0	179.8	0	X	X	F		Blue
Oyeye-RS	99		-1301.239	-1301.189	-1301.536	-33.3	252.6	269.0	172.9	0	X	X	F		Blue
Oyeye-SR	100		-1301.234	-1301.184	-1301.529	-33.0	252.5	269.3	176.9	0	X	X	F		Blue
Oyeye-SS	101		-1301.232	-1301.190	-1301.536	-28.5	252.7	269.2	173.8	0	X	X	F		Blue
oeeee	1		-468.238	-468.236	-468.484	-3.3	179.3	187.8	107.8	0	F	F	F		Red
oeeoe-R	4		-543.483	-543.473	-543.726	-8.1	183.1	192.0	111.5	0	F	F	I		Green
oeeoe-S	5		-543.481	-543.471	-543.724	-8.0	183.1	192.0	112.2	0	F	F	I		Green
oeeoo	11		-618.726	-618.707	-618.963	-13.8	186.5	196.1	117.0	0	X	X	X	I	Black
oeexe-R	18		-617.575	-617.558	-617.791	-12.6	171.5	180.7	115.3	0	X	F	F		Red
oeexe-S	19		-617.572	-617.556	-617.790	-12.1	171.6	180.8	114.0	0	X	F	F		Red
oeeye-R	14		-542.294	-542.283	-542.512	-8.6	167.7	176.4	110.4	0	X	X	I		Gray
oeeye-S	15		-542.294	-542.283	-542.511	-8.5	167.5	176.4	111.7	0	X	X	I		Gray
oeeyo-5A-RR	40		-617.534	-617.521	-617.758	-10.0	173.0	181.6	108.9	0	X	X	F		Blue
oeeyo-5A-RS	41		-617.537	-617.519	-617.756	-13.2	172.7	181.4	111.3	0	X	X	F		Blue
oeeyo-5A-SR	42		-617.532	-617.515	-617.752	-12.4	172.6	181.4	111.6	0	X	X	F		Blue
oeeyo-5A-SS	43		-617.530	-617.515	-617.751	-11.3	172.8	181.5	110.7	0	X	X	F		Blue
oeeyo-R	30		-617.534	-617.520	-617.753	-11.0	171.5	180.6	114.2	0	X	X	X	I	Black
oeeyo-S	31		-617.533	-617.518	-617.752	-11.6	171.5	180.7	114.5	0	X	X	X	I	Black
oeeyy	50		-616.342	-616.327	-616.534	-11.4	155.5	164.8	116.3	0	X	X	F		Blue
oeoee	3		-543.490	-543.480	-543.733	-7.9	182.5	191.4	109.6	0	F	F	I		Green
oeoee-4D	51		-467.003	-467.000	-467.228	-3.7	165.2	172.8	100.5	0	I	X	X		Gray
oeooe-R	9		-618.730	-618.716	-618.973	-10.3	185.9	195.4	115.1	0	X	X	X	I	Black
oeooe-S	10		-618.730	-618.711	-618.967	-13.6	185.7	195.3	116.1	0	X	X	X	I	Black
oeoye-5A-RR	36		-617.539	-617.525	-617.760	-10.8	171.7	180.6	110.1	0	X	X	F		Blue
oeoye-5A-RS	37		-617.539	-617.528	-617.764	-8.9	171.9	180.7	109.3	0	X	X	F		Blue
oeoye-5A-SR	38		-617.539	-617.526	-617.762	-9.8	172.0	180.7	109.6	0	X	X	F		Blue
oeoye-5A-SS	39		-617.538	-617.523	-617.759	-11.3	171.9	180.6	110.3	0	X	X	F		Blue
oeoye-R	28		-617.535	-617.523	-617.755	-9.1	170.2	179.6	114.9	0	X	X	X	I	Black
oeoye-S	29		-617.538	-617.522	-617.754	-11.4	170.0	179.6	115.7	0	X	X	X	I	Black
ooeee	2		-543.483	-543.470	-543.721	-10.2	182.5	191.8	114.3	0	X	F	I		Green
ooeoe-R	7		-618.726	-618.705	-618.963	-14.9	186.8	196.3	116.3	0	X	X	F		Blue
ooeoe-S	8		-618.724	-618.704	-618.960	-14.5	186.4	196.0	117.6	0	X	X	F		Blue
ooeye-5A-RR	32		-617.533	-617.515	-617.752	-13.2	173.0	181.7	110.6	0	X	X	F		Blue
ooeye-5A-RS	33		-617.532	-617.514	-617.751	-12.9	172.9	181.5	111.1	0	X	X	F		Blue
ooeye-5A-SR	34		-617.534	-617.518	-617.754	-12.2	172.6	181.4	111.3	0	X	X	F		Blue
ooeye-5A-SS	35		-617.536	-617.519	-617.756	-12.6	172.7	181.4	111.0	0	X	X	F		Blue
ooeye-R	26		-617.538	-617.517	-617.749	-15.3	170.8	180.3	116.9	0	X	X	X	I	Black
ooeye-S	27		-617.538	-617.517	-617.748	-15.3	170.6	180.2	118.5	0	X	X	X	I	Black
oooee	6		-618.734	-618.716	-618.971	-13.7	185.5	195.2	116.5	0	X	F	X		Green
oooee-4D	52		-542.248	-542.234	-542.465	-10.4	168.4	176.8	106.8	0	X	X	X	I	Black
oxeee-R	16		-617.572	-617.550	-617.781	-15.8	171.2	180.6	118.2	0	X	F	F		Red
oxeee-S	17		-617.569	-617.548	-617.780	-15.1	171.2	180.6	118.5	0	X	F	F		Red
oxoee-4D-R	55		-616.335	-616.314	-616.525	-15.2	156.9	165.5	110.3	0	F	X	X		Green
oxoee-4D-S	56		-616.329	-616.307	-616.518	-15.7	156.9	165.6	111.2	0	F	X	X		Green
oxoee-R	44		-692.823	-692.793	-693.029	-20.8	174.2	184.1	120.4	0	F	F	X		Green
oxoee-S	45		-692.820	-692.791	-693.028	-20.0	174.4	184.1	118.9	0	F	F	X		Green
oyeee-R	12		-542.292	-542.281	-542.509	-8.6	167.4	176.3	111.9	0	X	X	I		Gray
oyeee-S	13		-542.290	-542.280	-542.508	-8.1	167.7	176.5	111.1	0	X	X	I		Gray
oyeoe-RR	22		-617.533	-617.516	-617.748	-12.7	171.1	180.5	116.3	0	X	X	X	I	Black
oyeoe-RS	23		-617.534	-617.516	-617.748	-13.3	171.1	180.4	117.0	0	X	X	X	I	Black
oyeoe-SR	24		-617.531	-617.513	-617.747	-12.7	171.4	180.7	115.4	0	X	X	X	I	Black
oyeoe-SS	25		-617.532	-617.513	-617.746	-13.5	171.3	180.5	114.9	0	X	X	X	I	Black
oyeye-RR	46		-616.346	-616.328	-616.534	-13.3	155.4	164.8	118.9	0	X	X	F		Blue
oyeye-RS	47		-616.347	-616.328	-616.536	-14.2	155.7	164.9	116.0	0	X	X	F		Blue
oyeye-SR	48		-616.343	-616.326	-616.534	-12.5	155.8	165.0	116.0	0	X	X	F		Blue
oyeye-SS	49		-616.344	-616.325	-616.532	-13.7	155.3	164.7	117.6	0	X	X	F		Blue
oyoee-4D-R	53		-541.057	-541.045	-541.252	-9.1	153.3	161.3	105.3	0	X	X	X	I	Black
oyoee-4D-S	54		-541.055	-541.044	-541.251	-9.0	153.4	161.3	104.6	0	X	X	X	I	Black
oyoee-R	20		-617.543	-617.525	-617.757	-13.4	170.7	179.9	114.0	0	X	X	X	I	Black

oyoee-S	**21**		-617.542	-617.525	-617.757	-12.7	170.6	179.9	114.4	0	X	X	X	I	**Black**
seeee	**57**		-1092.129	-1092.116	-1092.373	-9.9	188.9	199.9	130.2	0	X	X	F		**Blue**
seeoe-R	**59**		-1167.367	-1167.344	-1167.606	-16.0	192.3	203.9	133.2	0	X	X	F		**Blue**
seeoe-S	**60**		-1167.370	-1167.349	-1167.608	-15.2	191.8	203.6	137.3	0	X	X	F		**Blue**
soeee	**58**		-1167.372	-1167.348	-1167.608	-16.9	192.0	203.8	137.5	0	X	X	F		**Blue**
soeoe-R	**61**		-1242.606	-1242.575	-1242.840	-21.6	195.6	207.8	139.5	0	X	X	F		**Blue**
soeoe-S	**62**		-1242.607	-1242.576	-1242.840	-21.6	195.4	207.8	141.4	0	X	X	F		**Blue**
										X	93	84	30		
										F	8	12	64		
										I	1	6	8	24	
										**Red**					**5**
										**Green**					**9**
										**Blue**					**59**
										**Gray**					**5**
										**Black**					**24**
										Total	102	102	102	24	102

**Hess's law**						
**Reactions**	**ΔG^*_{soln}**	**$\Delta G°_{gas}$**	**$\Delta\Delta G_{solv}+\Delta\Delta G$ o->***	**STEP**	**RXN**	**POS**
	**kcal/mol**	**kcal/mol**	**kcal/mol**			
C6H10O7 -> H2O	429778.3	429754.9	23.4			
2H3O+ + SO4-2 -> 3H2O	391473.2	391061.7	411.5			
1/2O2 -> 0O2	47165.0	47164.9	0.1			
0H2 -> H2	-737.6	-741.1	3.5			
0HO2 -> H2O	-47980.5	-47976.2	-4.3			
oeeee + C6H10O7 -> H2O + Oeeee	3.0	-2.9	5.9	1	g	3
ooeee + C6H10O7 -> H2O + Ooeee	3.4	-0.1	3.6	2	g	3
ooeee + C6H10O7 -> H2O + oOeee	2.2	-1.7	3.9	2	g	7
oeoee + C6H10O7 -> H2O + Oeoee	1.2	-3.7	4.9	2	g	3
oeoee + C6H10O7 -> H2O + oeOee	8.3	1.6	6.7	2	g	8
oeeoe-R + C6H10O7 -> H2O + Oeeoe-R	4.9	-1.6	6.5	2	g	3
oeeoe-S + C6H10O7 -> H2O + Oeeoe-S	7.5	-1.6	9.1	2	g	3
oeeoe-R + C6H10O7 -> H2O + oeeOe-R	5.7	-0.3	6.0	2	g	9
oeeoe-S + C6H10O7 -> H2O + oeeOe-S	2.3	-4.9	7.2	2	g	9
oyeee-R + C6H10O7 -> H2O + Oyeee-R	3.0	-0.3	3.3	3	g	3
oyeee-S + C6H10O7 -> H2O + Oyeee-S	1.9	-1.2	3.1	3	g	3
oeeye-R + C6H10O7 -> H2O + Oeeye-R	1.2	-7.7	8.9	3	g	3
oeeye-S + C6H10O7 -> H2O + Oeeye-S	1.5	-7.2	8.7	3	g	3
ooeoe-R + C6H10O7 -> H2O + Ooeoe-R	7.9	4.7	3.2	3	g	3
ooeoe-S + C6H10O7 -> H2O + Ooeoe-S	10.7	8.1	2.6	3	g	3
ooeoe-R + C6H10O7 -> H2O + oOeoe-R	0.8	-3.1	3.9	3	g	7
ooeoe-S + C6H10O7 -> H2O + oOeoe-S	2.7	-0.9	3.6	3	g	7
ooeoe-R + C6H10O7 -> H2O + ooeOe-R	2.1	0.0	2.1	3	g	9
ooeoe-S + C6H10O7 -> H2O + ooeOe-S	1.8	-1.5	3.3	3	g	9
oxeee-R + C6H10O7 -> H2O + Oxeee-R	7.4	3.9	3.4	4	g	3
oxeee-S + C6H10O7 -> H2O + Oxeee-S	3.1	-1.7	4.9	4	g	3
oeexe-R + C6H10O7 -> H2O + Oeexe-R	5.8	2.5	3.3	4	g	3
oeexe-S + C6H10O7 -> H2O + Oeexe-S	4.9	2.1	2.8	4	g	3
oxeee-R + C6H10O7 -> H2O + oXeee-R	15.0	10.3	4.7	4	g	7
oxeee-S + C6H10O7 -> H2O + oXeee-S	15.1	10.2	4.9	4	g	7
oeexe-R + C6H10O7 -> H2O + oeeXe-R	9.3	5.2	4.1	4	g	9
oeexe-S + C6H10O7 -> H2O + oeeXe-S	10.0	4.6	5.3	4	g	9
ooeye-5A-RR + C6H10O7 -> H2O + oOeye-5A-RR	1.5	-2.5	3.9	5	g	7
ooeye-5A-RS + C6H10O7 -> H2O + oOeye-5A-RS	1.3	-2.2	3.5	5	g	7
ooeye-5A-SR + C6H10O7 -> H2O + oOeye-5A-SR	1.3	-1.6	2.9	5	g	7
ooeye-5A-SS + C6H10O7 -> H2O + oOeye-5A-SS	2.6	-0.3	2.9	5	g	7
ooeye-5A-RS + C6H10O7 -> H2O + ooeYe-5A-RR	7.8	5.4	2.4	5	g	9
ooeye-5A-RR + C6H10O7 -> H2O + ooeYe-5A-RS	7.5	1.1	6.4	5	g	9
ooeye-5A-SS + C6H10O7 -> H2O + ooeYe-5A-SR	2.3	-1.1	3.5	5	g	9
ooeye-5A-SR + C6H10O7 -> H2O + ooeYe-5A-SS	6.3	2.7	3.6	5	g	9
oyeye-RR + C6H10O7 -> H2O + Oyeye-RR	8.2	3.3	5.0	5	g	3
oyeye-RS + C6H10O7 -> H2O + Oyeye-RS	2.3	-2.0	4.3	5	g	3
oyeye-SR + C6H10O7 -> H2O + Oyeye-SR	3.0	0.1	2.9	5	g	3
oyeye-SS + C6H10O7 -> H2O + Oyeye-SS	4.3	-4.3	8.6	5	g	3
oeeyy + C6H10O7 -> H2O + Oeeyy	7.7	4.0	3.7	5	g	3
oeeee + 2H3O+ + SO4-2 -> 3H2O + seeee	-24.0	-428.9	404.9	1	s	3
ooeee + 2H3O+ + SO4-2 -> 3H2O + soeee	-23.0	-427.7	404.7	2	s	3
oeeoe-R + 2H3O+ + SO4-2 -> 3H2O + seeoe-R	-19.9	-423.5	403.6	2	s	3
oeeoe-S + 2H3O+ + SO4-2 -> 3H2O + seeoe-S	-23.4	-427.7	404.3	2	s	3
ooeoe-R + 2H3O+ + SO4-2 -> 3H2O + soeoe-R	-17.5	-422.3	404.8	3	s	3
ooeoe-S + 2H3O+ + SO4-2 -> 3H2O + soeoe-S	-19.2	-423.6	404.4	3	s	3
oeeee + 1/2O2 -> ooeee	-52.1	-45.3	-6.8	1	o1	7
oeeee + 1/2O2 -> oeoee	-56.0	-51.5	-4.5	1	o1	8
oeeee + 1/2O2 -> oeeoe-R	-51.9	-47.2	-4.8	1	o1	9
oeeee + 1/2O2 -> oeeoe-S	-50.4	-45.7	-4.7	1	o1	9
Oeeee + 1/2O2 -> Ooeee	-51.7	-42.5	-9.2	2	o1	7
Oeeee + 1/2O2 -> Oeoee	-57.8	-52.3	-5.5	2	o1	8
Oeeee + 1/2O2 -> Oeeoe-R	-50.0	-45.8	-4.2	2	o1	9
Oeeee + 1/2O2 -> Oeeoe-S	-45.9	-44.4	-1.4	2	o1	9
ooeee + 1/2O2 -> oooee	-55.7	-52.3	-3.5	2	o1	8
oeoee + 1/2O2 -> oooee	-51.8	-46.1	-5.7	2	o1	7
ooeee + 1/2O2 -> ooeoe-R	-50.5	-45.9	-4.7	2	o1	9
ooeee + 1/2O2 -> ooeoe-S	-49.3	-45.0	-4.3	2	o1	9
oeeoe-R + 1/2O2 -> ooeoe-R	-50.7	-44.0	-6.7	2	o1	7
oeeoe-S + 1/2O2 -> ooeoe-S	-51.0	-44.6	-6.4	2	o1	7
oeoee + 1/2O2 -> oeooe-R	-48.8	-46.5	-2.3	2	o1	9
oeoee + 1/2O2 -> oeooe-S	-48.9	-43.3	-5.7	2	o1	9
oeeoe-S + 1/2O2 -> oeooe-R	-54.5	-52.3	-2.2	2	o1	8
oeeoe-R + 1/2O2 -> oeooe-S	-53.0	-47.6	-5.4	2	o1	8
oeeoe-R + 1/2O2 -> oeeoo	-50.5	-44.9	-5.6	2	o1	10
oeeoe-S + 1/2O2 -> oeeoo	-52.0	-46.3	-5.7	2	o1	10
Ooeee + 1/2O2 -> Ooeoe-R	-46.1	-41.1	-5.0	3	o1	9
Ooeee + 1/2O2 -> Ooeoe-S	-42.0	-36.8	-5.2	3	o1	9
Oeeoe-R + 1/2O2 -> Ooeoe-R	-47.8	-37.8	-10.0	3	o1	7
Oeeoe-S + 1/2O2 -> Ooeoe-S	-47.8	-34.9	-12.9	3	o1	7
oOeee + 1/2O2 -> oOeoe-R	-52.0	-47.3	-4.7	3	o1	9
oOeee + 1/2O2 -> oOeoe-S	-48.8	-44.2	-4.6	3	o1	9
oeeOe-R + 1/2O2 -> ooeOe-R	-54.4	-43.7	-10.6	3	o1	7
oeeOe-S + 1/2O2 -> ooeOe-S	-51.5	-41.2	-10.4	3	o1	7
oeoee-4D + 1/2O2 -> oooee-4D	-51.9	-45.2	-6.7	3	o1	7
oyeee-R + 1/2O2 -> oyoee-R	-55.8	-51.0	-4.7	3	o1	8
oyeee-S + 1/2O2 -> oyoee-S	-56.4	-51.8	-4.5	3	o1	8
oyeee-R + 1/2O2 -> oyeoe-RR	-49.5	-45.5	-4.0	3	o1	9
oyeee-R + 1/2O2 -> oyeoe-RS	-50.3	-45.6	-4.7	3	o1	9
oyeee-S + 1/2O2 -> oyeoe-SR	-49.4	-44.9	-4.5	3	o1	9
oyeee-S + 1/2O2 -> oyeoe-SS	-50.1	-44.8	-5.3	3	o1	9
oeeye-R + 1/2O2 -> ooeye-R	-51.6	-44.9	-6.7	3	o1	7
oeeye-S + 1/2O2 -> ooeye-S	-51.8	-45.0	-6.7	3	o1	7
oeeye-S + 1/2O2 -> oeoye-R	-49.5	-48.9	-0.6	3	o1	8
oeeye-R + 1/2O2 -> oeoye-S	-51.0	-48.2	-2.8	3	o1	8
oeeye-R + 1/2O2 -> oeeyo-R	-49.0	-46.7	-2.4	3	o1	10
oeeye-S + 1/2O2 -> oeeyo-S	-48.7	-45.7	-3.0	3	o1	10
oxeee-S + 1/2O2 -> oxoee-S	-55.3	-50.5	-4.8	4	o1	8
oxeee-R + 1/2O2 -> oxoee-R	-55.9	-51.0	-4.9	4	o1	8
oyeee-R + 1/2O2 -> oxeee-R	-73.8	-66.7	-7.1	3	o2	7
oyeee-S + 1/2O2 -> oxeee-S	-73.6	-66.8	-6.9	3	o2	7
oeeye-R + 1/2O2 -> oeexe-R	-74.3	-70.4	-3.9	3	o2	9

oeeye-S + 1/2O2 -> oeexe-S	-72.8	-69.3				-3.5				3	o2	9
Oyeee-R + 1/2O2 -> Oxeee-R	-69.4	-62.5				-6.9				4	o2	7
Oyeee-S + 1/2O2 -> Oxeee-S	-72.4	-67.3				-5.1				4	o2	7
Oeeye-R + 1/2O2 -> Oeexe-R	-69.8	-60.2				-9.6				4	o2	9
Oeeye-S + 1/2O2 -> Oeexe-S	-69.4	-60.0				-9.4				4	o2	9
oyoee-R + 1/2O2 -> oxoee-R	-74.0	-66.7				-7.3				4	o2	7
oyoee-S + 1/2O2 -> oxoee-S	-72.6	-65.4				-7.2				4	o2	7
oyoee-4D-R + 1/2O2 -> oxoee-4D-R	-72.9	-67.0				-6.0				5	o2	7
oyoee-4D-S + 1/2O2 -> oxoee-4D-S	-70.2	-63.5				-6.7				5	o2	7
ooeee -> oyeee-R + H2	10.1	5.0				5.1				2	o3	7
ooeee -> oyeee-S + H2	11.4	5.8				5.6				2	o3	7
oeeoe-R -> oeeye-R + H2	8.5	5.4				3.1				2	o3	9
oeeoe-S -> oeeye-S + H2	7.2	4.1				3.1				2	o3	9
Ooeee -> Oyeee-R + H2	9.7	4.8				4.8				3	o3	7
Ooeee -> Oyeee-S + H2	9.9	4.8				5.1				3	o3	7
Oeeoe-R -> Oeeye-R + H2	4.8	-0.7				5.5				3	o3	9
Oeeoe-S -> Oeeye-S + H2	1.2	-1.4				2.6				3	o3	9
oooee -> oyoee-R + H2	10.1	6.3				3.8				3	o3	7
oooee -> oyoee-S + H2	10.7	6.2				4.5				3	o3	7
ooeoe-R -> oyeoe-RR + H2	11.2	5.4				5.8				3	o3	7
ooeoe-R -> oyeoe-RS + H2	10.4	5.2				5.1				3	o3	7
ooeoe-S -> oyeoe-SR + H2	11.2	5.9				5.3				3	o3	7
ooeoe-S -> oyeoe-SS + H2	10.5	6.0				4.5				3	o3	7
ooeoe-R -> ooeye-R + H2	7.7	4.5				3.2				3	o3	9
ooeoe-S -> ooeye-S + H2	6.4	3.7				2.7				3	o3	9
oeooe-R -> oeoye-R + H2	12.2	7.5				4.7				3	o3	9
oeooe-S -> oeoye-S + H2	10.5	4.8				5.7				3	o3	9
oeeoo -> oeeyo-R + H2	9.9	3.6				6.3				3	o3	9
oeeoo -> oeeyo-S + H2	10.5	4.8				5.7				3	o3	9
oooee-4D -> oyoee-4D-R + H2	9.9	5.1				4.8				4	o3	7
oooee-4D -> oyoee-4D-S + H2	11.0	6.0				5.0				4	o3	7
oyeoe-RR -> oyeye-RR + H2	7.2	4.3				2.9				4	o3	9
oyeoe-RS -> oyeye-RS + H2	7.1	4.4				2.7				4	o3	9
oyeoe-SR -> oyeye-SR + H2	7.6	3.8				3.8				4	o3	9
oyeoe-SS -> oyeye-SS + H2	8.0	4.6				3.3				4	o3	9
ooeye-R -> oyeye-RR + H2	10.7	5.2				5.5				4	o3	7
ooeye-R -> oyeye-SR + H2	12.4	6.1				6.3				4	o3	7
ooeye-S -> oyeye-RS + H2	9.8	5.1				4.7				4	o3	7
ooeye-S -> oyeye-SS + H2	12.1	7.0				5.1				4	o3	7
oeeyo-R -> oeeyy + H2	10.6	7.4				3.2				4	o3	10
oeeyo-S -> oeeyy + H2	10.0	6.3				3.7				4	o3	10
oeoee -> oeoee-4D + H2O	15.6	15.6				-0.1				2	d	
oooee -> oooee-4D + H2O	15.5	16.6				-1.1				3	d	
oyoee-R -> oyoee-4D-R + H2O	15.3	15.4				-0.1				4	d	
oyoee-S -> oyoee-4D-S + H2O	15.8	16.3				-0.6				4	d	
oxoee-R -> oxoee-4D-R + H2O	16.3	15.1				1.2				5	d	
oxoee-S -> oxoee-4D-S + H2O	18.1	18.2				-0.1				5	d	
ooeye-R -> ooeye-5A-RR	3.3	1.2				2.1				4	a	
ooeye-R -> ooeye-5A-RS	4.2	1.8				2.4				4	a	
ooeye-S -> ooeye-5A-SR	2.8	-0.4				3.1				4	a	
ooeye-S -> ooeye-5A-SS	1.2	-1.5				2.7				4	a	
oeoye-R -> oeoye-5A-RR	-2.7	-1.0				-1.6				4	a	
oeoye-R -> oeoye-5A-RS	-2.6	-2.9				0.2				4	a	
oeoye-S -> oeoye-5A-SR	-0.7	-2.3				1.6				4	a	
oeoye-S -> oeoye-5A-SS	-0.5	-0.6				0.1				4	a	
oeeyo-R -> oeeyo-5A-RR	0.5	-0.5				1.0				4	a	
oeeyo-R -> oeeyo-5A-RS	-1.9	0.3				-2.2				4	a	
oeeyo-S -> oeeyo-5A-SR	0.8	1.7				-0.9				4	a	
oeeyo-S -> oeeyo-5A-SS	2.3	2.0				0.3				4	a	

Step of reaction	Average		SD	
	ΔG^*_{soln}	$\Delta G°_{gas}$	ΔG^*_{soln}	$\Delta G°_{gas}$
	kcal/mol	kcal/mol	kcal/mol	kcal/mol
Step 1	-38.6	-103.6	23.4	160.4
Step 2	-25.0	-62.5	28.2	121.2
Step 3	-22.0	-36.6	31.1	80.9
Step 4	-7.5	-8.4	30.0	26.0
Step 5	-3.1	-5.6	26.2	23.3

Type of reaction	Average		SD	
	ΔG^*_{soln}	$\Delta G°_{gas}$	ΔG^*_{soln}	$\Delta G°_{gas}$
	kcal/mol	kcal/mol	kcal/mol	kcal/mol
g	5.0	0.4	3.7	4.1
s	-21.2	-425.6	2.7	2.8
o1	-51.1	-45.8	3.1	3.9
o2	-72.1	-65.5	1.9	3.3
o3	9.4	4.9	2.3	1.9
d	16.1	16.2	1.0	1.1
a	0.6	-0.2	2.3	1.6

Position of reaction	Average		SD	
	ΔG^*_{soln}	$\Delta G°_{gas}$	ΔG^*_{soln}	$\Delta G°_{gas}$
	kcal/mol	kcal/mol	kcal/mol	kcal/mol
3	-1.3	-98.4	11.4	182.8
7	-21.8	-20.9	35.2	29.3
8	-49.4	-46.3	18.3	15.2
9	-20.6	-20.7	31.1	26.6
10	-29.9	-28.3	31.2	27.2

Type and position of reaction	Average		SD	
	ΔG^*_{soln}	$\Delta G°_{gas}$	ΔG^*_{soln}	$\Delta G°_{gas}$
	kcal/mol	kcal/mol	kcal/mol	kcal/mol
g-3	4.7	-0.3	2.8	4.0
g-7	4.7	0.9	5.9	5.4
g-8	8.3	1.6	#DIV/0!	#DIV/0!
g-9	5.5	1.1	3.2	3.4
s-3	-21.2	-425.6	2.7	2.8
o1-7	-51.2	-42.9	1.8	0.6
o1-8	-54.6	-50.7	2.5	1.7
o1-9	-49.0	-44.6	2.5	2.6
o1-10	-50.0	-45.9	1.5	0.8
o2-7	-72.4	-65.7	1.7	1.8
o2-9	-71.6	-65.0	2.4	5.6
o3-7	10.7	5.6	0.8	0.6
o3-9	7.8	3.8	2.7	2.3
o3-10	10.3	6.8	0.4	0.8
4D	16.1	16.2	1.0	1.1
5A	0.6	-0.2	2.3	1.6

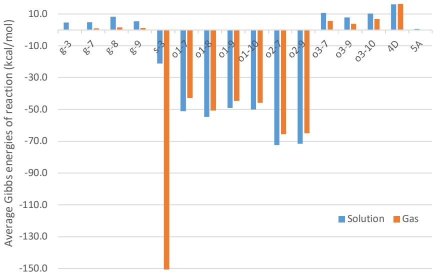
Average Gibbs energies of reaction (kcal/mol)
10.0
-10.0
-30.0
-50.0
-70.0
-90.0
-110.0
-130.0
-150.0
g-3
g-7
g-8
g-9
s-3
o1-7
o1-8
o1-9
o1-10
o2-7
o2-9
o3-7
o3-9
o3-10
4D
5A
Solution
Gas

name	step	energy (kcal/mol)		reactions
		aqueous phase	gas phase	
oeeee -> oeeee	0	0.0	0.0	
oeeee + 1/2O2 -> ooeee	1	-52.1	-45.3	o1
oeeee + 1/2O2 -> oeoee	1	-56.0	-51.5	
oeeee + 1/2O2 -> oeeoe-R	1	-51.9	-47.2	
oeeee + 1/2O2 -> oeeoe-S	1	-50.4	-45.7	
oeeee + C6H10O7 -> H2O + Oeeee	1	3.0	-2.9	g
oeeee + 2H3O+ + SO4-2 -> 3H2O + seeee	1	-24.0	-428.9	s
oeeee + O2 -> oooee	2	-107.9	-97.6	o1 x 2
oeeee + O2 -> ooeoe-R	2	-102.7	-91.2	
oeeee + O2 -> ooeoe-S	2	-101.4	-90.3	
oeeee + O2 -> oeooe-R	2	-104.9	-98.0	
oeeee + O2 -> oeooe-S	2	-105.0	-94.8	
oeeee + O2 -> oeeoo	2	-102.4	-92.0	
oeeee + 1/2O2 -> oeoee-4D + H2O	2	-40.5	-35.8	o1, 4D
oeeee + 1/2O2 -> oyeee-R + H2	2	-42.0	-40.3	o1, o3
oeeee + 1/2O2 -> oyeee-S + H2	2	-40.7	-39.5	
oeeee + 1/2O2 -> oeeye-R + H2	2	-43.4	-41.7	
oeeee + 1/2O2 -> oeeye-S + H2	2	-43.2	-41.6	
oeeee + C6H10O7 + 1/2O2 -> H2O + Ooeee	2	-48.7	-45.4	o1, g
oeeee + C6H10O7 + 1/2O2 -> H2O + oOeee	2	-49.9	-47.0	
oeeee + C6H10O7 + 1/2O2 -> H2O + Oeoee	2	-54.8	-55.2	
oeeee + C6H10O7 + 1/2O2 -> H2O + oeOee	2	-47.7	-49.9	
oeeee + C6H10O7 + 1/2O2 -> H2O + Oeeoe-R	2	-47.0	-48.7	
oeeee + C6H10O7 + 1/2O2 -> H2O + Oeeoe-S	2	-42.9	-47.3	
oeeee + C6H10O7 + 1/2O2 -> H2O + oeeOe-R	2	-46.2	-47.4	
oeeee + C6H10O7 + 1/2O2 -> H2O + oeeOe-S	2	-48.1	-50.7	
oeeee + 2H3O+ + SO4-2 + 1/2O2 -> 3H2O + soeee	2	-75.1	-473.0	o1, s
oeeee + 2H3O+ + SO4-2 + 1/2O2 -> 3H2O + seeoe-R	2	-71.8	-470.6	
oeeee + 2H3O+ + SO4-2 + 1/2O2 -> 3H2O + seeoe-S	2	-73.8	-473.4	
oeeee + O2 -> H2 + oxeee-R	3	-115.8	-107.0	o1, o3, o2
oeeee + O2 -> H2 + oxeee-S	3	-114.4	-106.3	
oeeee + O2 -> H2 + oeexe-R	3	-117.7	-112.1	
oeeee + O2 -> H2 + oeexe-S	3	-116.0	-110.9	
oeeee + O2 -> H2 + oyoee-R	3	-97.8	-91.3	o1 x 2, o3
oeeee + O2 -> H2 + oyoee-S	3	-97.1	-91.3	
oeeee + O2 -> H2 + oyeoe-RR	3	-91.5	-85.8	
oeeee + O2 -> H2 + oyeoe-RS	3	-92.3	-85.9	
oeeee + O2 -> H2 + oyeoe-SR	3	-90.1	-84.4	
oeeee + O2 -> H2 + oyeoe-SS	3	-90.9	-84.3	
oeeee + O2 -> H2 + ooeye-R	3	-95.0	-86.6	
oeeee + O2 -> H2 + ooeye-S	3	-95.0	-86.6	
oeeee + O2 -> H2 + oeoye-R	3	-92.7	-90.5	
oeeee + O2 -> H2 + oeoye-S	3	-94.4	-90.0	
oeeee + O2 -> H2 + oeeyo-R	3	-92.4	-88.4	
oeeee + O2 -> H2 + oeeyo-S	3	-91.9	-87.3	
oeeee + O2 -> H2O + ooоee-4D	3	-92.3	-81.0	o1 x 2, 4D
oeeee + C6H10O7 + O2 -> H2O + Ooeoe-R	3	-94.8	-86.5	o1 x 2, g
oeeee + C6H10O7 + O2 -> H2O + Ooeoe-S	3	-90.7	-82.2	
oeeee + C6H10O7 + O2 -> H2O + oOeoe-R	3	-101.9	-94.3	
oeeee + C6H10O7 + O2 -> H2O + oOeoe-S	3	-98.7	-91.2	
oeeee + C6H10O7 + O2 -> H2O + ooeOe-R	3	-100.6	-91.2	
oeeee + C6H10O7 + O2 -> H2O + ooeOe-S	3	-99.6	-91.8	
oeeee + C6H10O7 + 1/2O2 -> H2O + H2 + Oyeee-R	3	-39.0	-40.6	o1, o3, g
oeeee + C6H10O7 + 1/2O2 -> H2O + H2 + Oyeee-S	3	-38.8	-40.7	
oeeee + C6H10O7 + 1/2O2 -> H2O + H2 + Oeeye-R	3	-42.2	-49.4	
oeeee + C6H10O7 + 1/2O2 -> H2O + H2 + Oeeye-S	3	-41.7	-48.8	
oeeee + 2H3O+ + SO4-2 + O2 -> 3H2O + soeoe-R	3	-120.1	-513.5	o1 x 2, s
oeeee + 2H3O+ + SO4-2 + O2 -> 3H2O + soeoe-S	3	-120.6	-514.0	
oeeee + 3/2O2 -> H2 + oxoee-R	4	-171.7	-158.0	o1 x 2, o3, o2
oeeee + 3/2O2 -> H2 + oxoee-S	4	-169.7	-156.7	
oeeee + O2 -> 2H2 + oyeye-RR	4	-84.3	-81.5	o1 x 2, o3 x 2
oeeee + O2 -> 2H2 + oyeye-RS	4	-85.2	-81.5	
oeeee + O2 -> 2H2 + oyeye-SR	4	-82.5	-80.5	
oeeee + O2 -> 2H2 + oyeye-SS	4	-82.9	-79.7	
oeeee + O2 -> 2H2 + oeeyy	4	-81.9	-81.0	
oeeee + O2 -> H2 + H2O + oyoee-4D-R	4	-82.5	-75.9	o1 x 2, o3, 4D
oeeee + O2 -> H2 + H2O + oyoee-4D-S	4	-81.4	-75.0	
oeeee + O2 -> H2 + ooeye-5A-RR	4	-91.7	-85.4	o1 x 2, o3, 5A
oeeee + O2 -> H2 + ooeye-5A-RS	4	-90.8	-84.9	

oeeee + O2 -> H2 + ooeye-5A-SR	4	-92.2	-87.0	
oeeee + O2 -> H2 + ooeye-5A-SS	4	-93.7	-88.1	
oeeee + O2 -> H2 + oeoye-5A-RR	4	-95.4	-91.6	
oeeee + O2 -> H2 + oeoye-5A-RS	4	-95.3	-93.4	
oeeee + O2 -> H2 + oeoye-5A-SR	4	-95.1	-92.2	
oeeee + O2 -> H2 + oeoye-5A-SS	4	-94.9	-90.5	
oeeee + O2 -> H2 + oeeyo-5A-RR	4	-92.0	-88.9	
oeeee + O2 -> H2 + oeeyo-5A-RS	4	-94.3	-88.1	
oeeee + O2 -> H2 + oeeyo-5A-SR	4	-91.1	-85.6	
oeeee + O2 -> H2 + oeeyo-5A-SS	4	-89.6	-85.2	
oeeee + C6H10O7 + O2 -> H2O + H2 + Oxeee-R	4	-108.4	-103.1	o1, o3, o2, g
oeeee + C6H10O7 + O2 -> H2O + H2 + Oxeee-S	4	-111.2	-108.0	
oeeee + C6H10O7 + O2 -> H2O + H2 + Oeexe-R	4	-112.0	-109.6	
oeeee + C6H10O7 + O2 -> H2O + H2 + Oeexe-S	4	-111.1	-108.8	
oeeee + C6H10O7 + O2 -> H2O + H2 + oXeee-R	4	-100.8	-96.7	
oeeee + C6H10O7 + O2 -> H2O + H2 + oXeee-S	4	-99.2	-96.0	
oeeee + C6H10O7 + O2 -> H2O + H2 + oeeXe-R	4	-108.4	-106.9	
oeeee + C6H10O7 + O2 -> H2O + H2 + oeeXe-S	4	-106.0	-106.2	
oeeee + 3/2O2 -> H2O + H2 + oxoee-4D-R	5	-155.4	-142.9	o1 x 2, o3, o2, 4D
oeeee + 3/2O2 -> H2O + H2 + oxoee-4D-S	5	-151.6	-138.5	
oeeee + C6H10O7 + O2 -> H2O + H2 + oOeye-5A-RR	5	-90.3	-87.9	o1 x 2, o3, g, 5A
oeeee + C6H10O7 + O2 -> H2O + H2 + oOeye-5A-RS	5	-89.5	-87.0	
oeeee + C6H10O7 + O2 -> H2O + H2 + oOeye-5A-SR	5	-90.9	-88.6	
oeeee + C6H10O7 + O2 -> H2O + H2 + oOeye-5A-SS	5	-91.1	-88.4	
oeeee + C6H10O7 + O2 -> H2O + H2 + ooeYe-5A-RR	5	-83.0	-79.5	
oeeee + C6H10O7 + O2 -> H2O + H2 + ooeYe-5A-RS	5	-84.2	-84.3	
oeeee + C6H10O7 + O2 -> H2O + H2 + ooeYe-5A-SR	5	-91.4	-89.2	
oeeee + C6H10O7 + O2 -> H2O + H2 + ooeYe-5A-SS	5	-85.9	-84.4	
oeeee + C6H10O7 + O2 -> H2O + 2H2 + Oyeye-RR	5	-76.1	-78.2	o1 x 2, o3 x 2, g
oeeee + C6H10O7 + O2 -> H2O + 2H2 + Oyeye-RS	5	-82.9	-83.5	
oeeee + C6H10O7 + O2 -> H2O + 2H2 + Oyeye-SR	5	-79.6	-80.4	
oeeee + C6H10O7 + O2 -> H2O + 2H2 + Oyeye-SS	5	-78.6	-83.9	
oeeee + C6H10O7 + O2 -> H2O + 2H2 + Oeeyy	5	-74.1	-77.0	

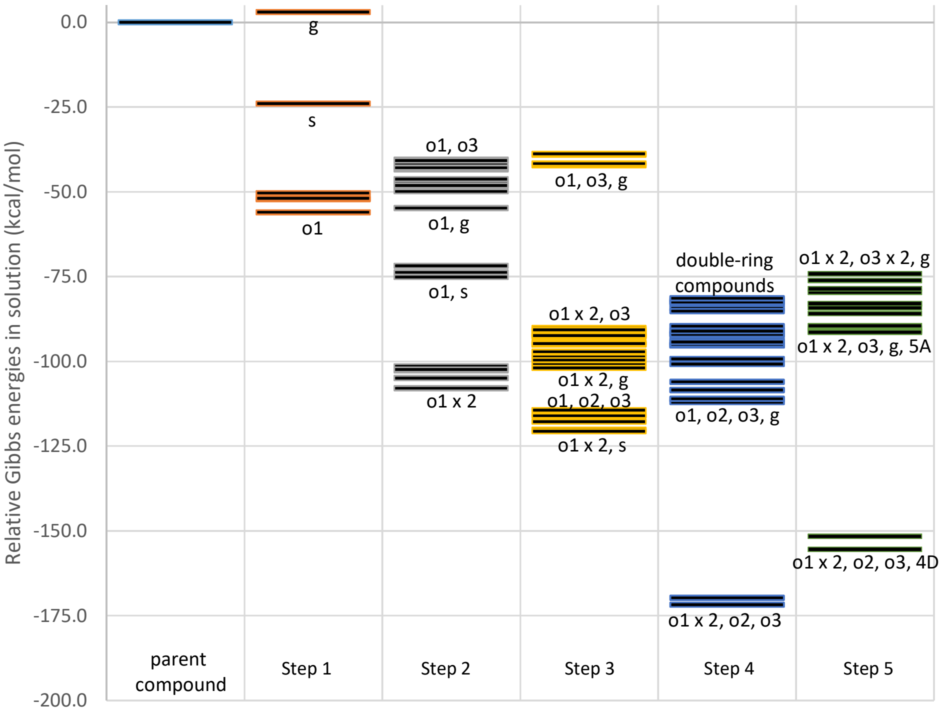

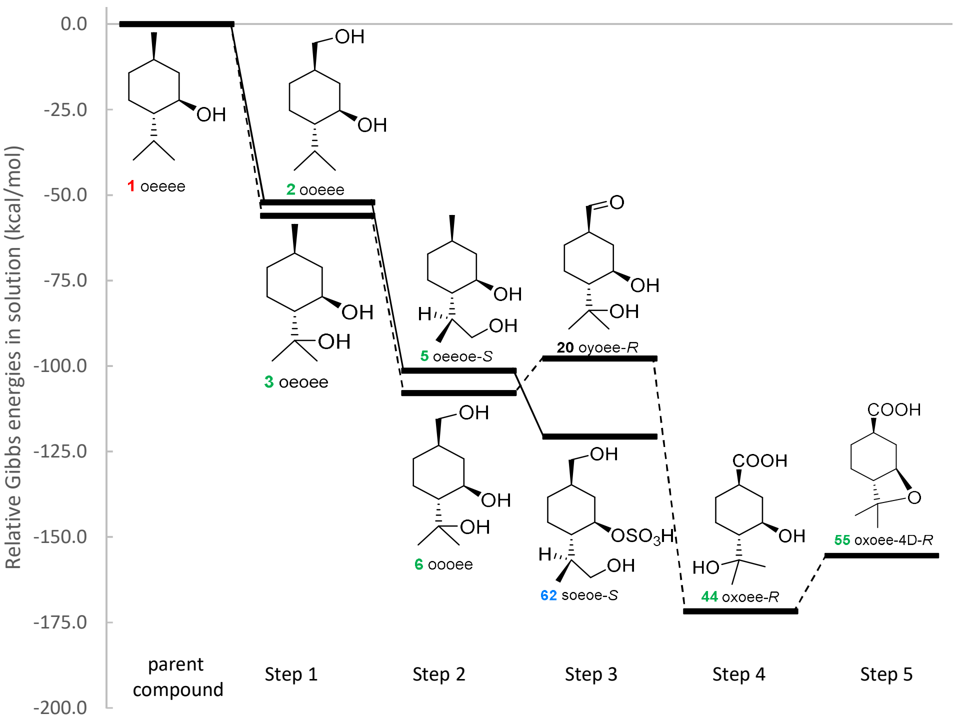

Relative Gibbs energies in solution (kcal/mol)
0.0
-25.0
-50.0
-75.0
-100.0
-125.0
-150.0
-175.0
-200.0
parent compound
Step 1
Step 2
Step 3
Step 4
Step 5
1 oeeee
2 ooeee
3 oeoee
5 oeeoe-S
6 oooee
20 oyoee-R
62 soeoe-S
44 oxoee-R
55 oxoee-4D-R
OH
OSO3H
COOH
HO
H
O

Name	Quantity	Unit	Ref
Hartree	627.50947	kcal mol^-1/AU	2014 CODATA

**Basis: 6-311++G(d,p)/Optimization in gas phase**	**Electronic Energy**					
**Compound**	**B3LYP**		**MP2**		**Difference**	
	**AU**	**kcal/mol**	**AU**	**kcal/mol**	**AU**	**kcal/mol**
H2	-1.1796		-1.1603		0.0193	
H2O	-76.4585		-76.2749		0.1836	
H3O+	-76.7312		-76.5501		0.1811	
O2	-150.3089		-149.9813		0.3277	
O4S-2	-699.1158		-697.8751		1.2407	
oeeee	-468.4839		-467.0444		1.4395	
oeoee	-543.7329		-542.1327		1.6003	
ooeee	-543.7214		-542.1178		1.6037	
oooee	-618.9713		-617.2073		1.7640	
ooeoe-S	-618.9605		-617.1930		1.7675	
oyoee-R	-617.7571		-616.0093		1.7478	
soeoe-S	-1242.8395		-1240.0348		2.8047	
oxoee-R	-693.0290		-691.1185		1.9105	
oxoee-4D-R	-616.5253		-614.7923		1.7330	
**Coefficient of determination**					**1.0000**	
**Slope**					**0.9976**	
**Intercept**					**0.1213**	
**Reaction**	**AU**	**kcal/mol**	**AU**	**kcal/mol**	**AU**	**kcal/mol**
oeeee + 1/2O2 -> oeoee	-0.0945	-59.3	-0.0976	-61.3		-1.9
oeeee + 1/2O2 -> ooeee	-0.0830	-52.1	-0.0827	-51.9		0.2
oeoee + 1/2O2 -> oooee	-0.0839	-52.6	-0.0840	-52.7		-0.1
ooeee + 1/2O2 -> oooee	-0.0953	-59.8	-0.0989	-62.0		-2.2
ooeee + 1/2O2 -> ooeoe-S	-0.0846	-53.1	-0.0846	-53.1		0.0
oooee -> oyoee-R + H2	0.0345	21.7	0.0377	23.6		2.0
ooeoe-S + 2H3O+ + SO4-2 -> 3H2O + soeoe-S	-0.6763	-424.4	-0.6912	-433.7		-9.3
oyoee-R + 1/2O2 -> oxoee-R	-0.1174	-73.7	-0.1186	-74.4		-0.7
oxoee-R -> oxoee-4D-R + H2O	0.0453	28.4	0.0513	32.2		3.8
**Coefficient of determination**						**0.9999**
**Slope**						**1.0259**
**Intercept**						**1.1613**
**Mean Absolute Error (MAE)**						**2.25**

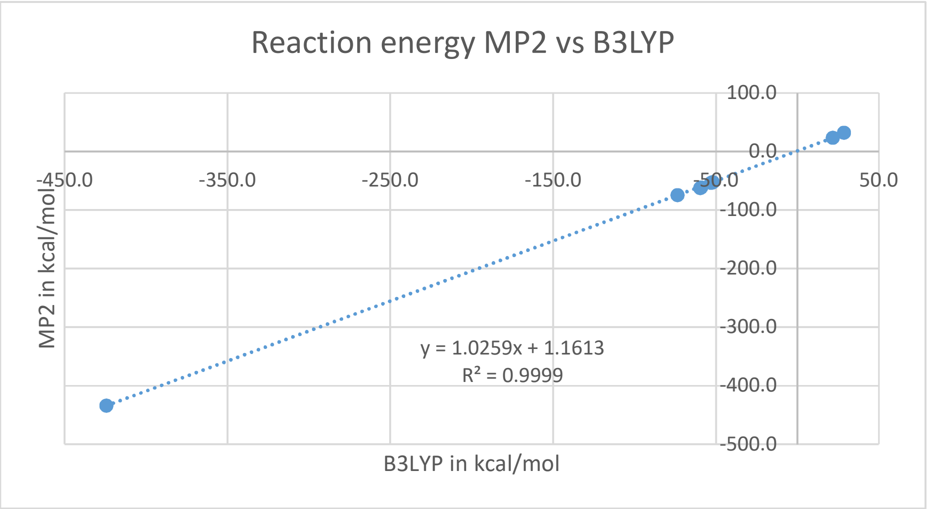

Supplement: S1 File — (ZIP) [file pone.0216577.s001.zip › calculations.pdf]
