# Supplementary material for: An in silico investigation of menthol metabolism
Source: PLoS One. 2019 Sep 27;14(9):e0216577. doi: 10.1371/journal.pone.0216577 (PMC6764827; doi:10.1371/journal.pone.0216577)

Abbreviations for the nomenclature of menthol metabolites referred to by the present study
and
a list of 102 compounds in this study grouped by molecular formula

Position / **Group**	3	7	8	9	10
Original form	o	e	e	e	e
Alkan**e**	-	e	e	e	e
Alcoh**o**l	o	o	o	o	o
Aldeh**y**de	y	y	-	y	y
Carbo**x**ylic acid	-	x	-	x	-
**D**ehydration	-4D for four-membered ring formation at positions 3 and 8				
**A**ldol reaction	-5A for four-membered ring formation at positions 3 and 9				
Glucuronic acid	O	O,X	-	O,X,Y	-
**S**ulfate group	s	-	-	-	-

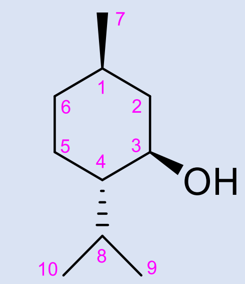

- An underlined indicates that there are *R* and *S* stereoisomers due to the substitution.
- Substitution at position 9 leads to a new chiral center if it is not the same as 10.
- Substitution at position 10 is forced to have lower or the same oxidation state for the carbon atom when compared to position 9.
- Dashes are where substitution with the functional group at that respective position cannot occur

formula: $C_{10}H_{20}O$ (molar mass 156.26, total 1 compound)

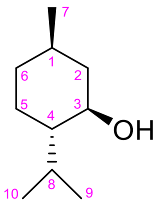

1 oeeee

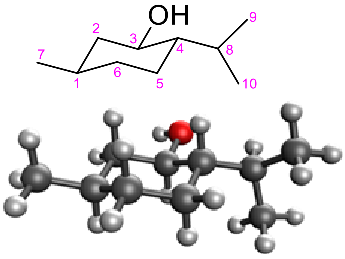

(in chair form)

formula: $C_{10}H_{20}O_2$ (molar mass 172.26, total 4 compounds)

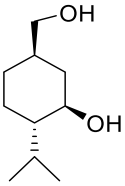

2 ooeee

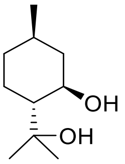

3 oeoee

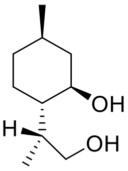

4 oeeoe-*R*

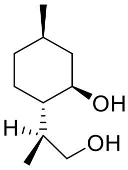

5 oeeoe-*S*

formula: $C_{10}H_{20}O_3$ (molar mass 188.26, total 6 compounds)

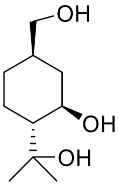

6 oooee

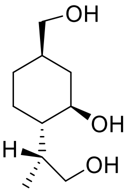

7 ooeoe-*R*

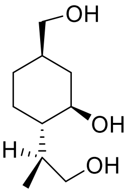

8 ooeoe-*S*

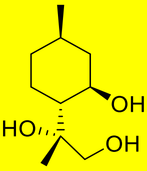

9 oeooe-*R*

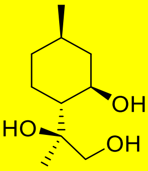

10 oeooe-*S*

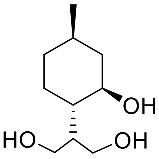

11 oeeoo

formula: $C_{10}H_{18}O_2$ (molar mass 170.25, total 4 compounds)

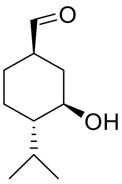

12 oyeee-*R*

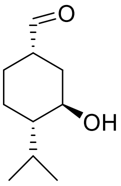

13 oyeee-*S*

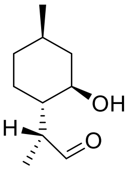

14 oeeye-*R*

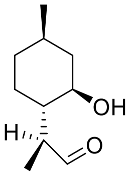

15 oeeye-*S*

formula: $C_{10}H_{18}O_3$ (molar mass 186.25, total 28 compounds)

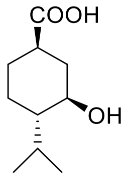
16 oxeee-*R*

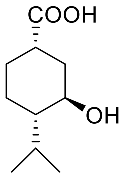
17 oxeee-*S*

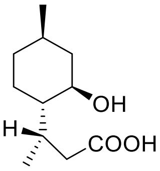
18 oeexe-*R*

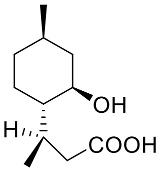
19 oeexe-*S*

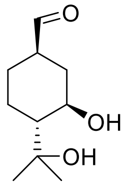
20 oyoee-*R*

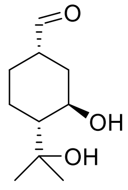
21 oyoee-*S*

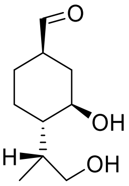
22 oyeoe-*RR*

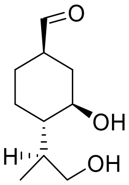
23 oyeoe-*RS*

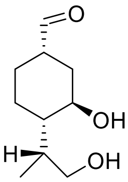
24 oyeoe-*SR*

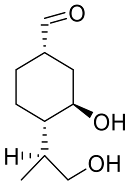
25 oyeoe-*SS*

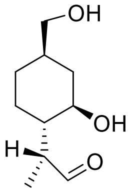
26 ooeye-*R*

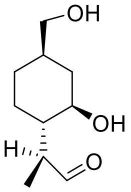
27 ooeye-*S*

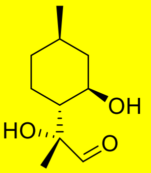
28 oeoye-*R*

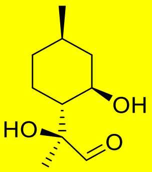
29 oeoye-*S*

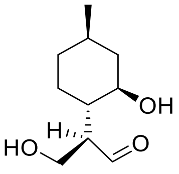
30 oeeyo-*R*

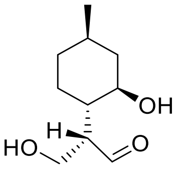
31 oeeyo-*S*

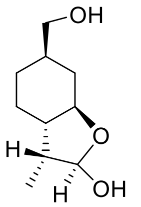
32 ooeye-5A-*RR*

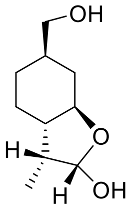
33 ooeye-5A-*RS*

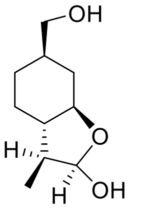
34 ooeye-5A-*SR*

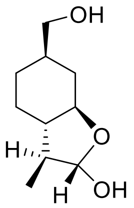
35 ooeye-5A-*SS*

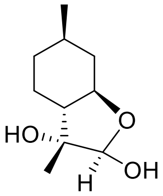
36 oeoye-5A-*RR*

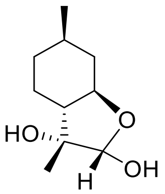
37 oeoye-5A-*RS*

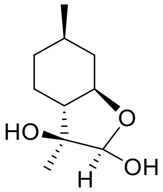
38 oeoye-5A-*SR*

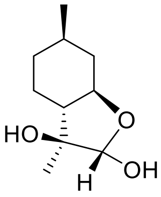
39 oeoye-5A-*SS*

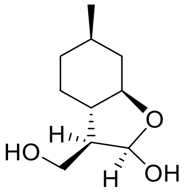
40 oeeyo-5A-*RR*

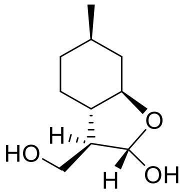
41 oeeyo-5A-*RS*

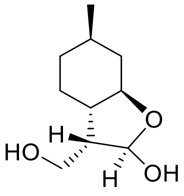
42 oeeyo-5A-*SR*

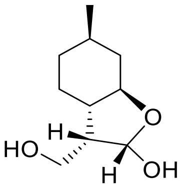
43 oeeyo-5A-*SS*

formula: $C_{10}H_{18}O_4$ (molar mass 202.25, total 2 compounds)

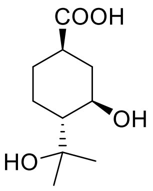
44 oxoee-*R*

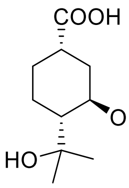
45 oxoee-*S*

formula: $C_{10}H_{16}O_3$ (molar mass 184.23, total 5 compounds)

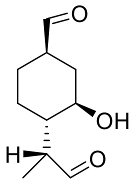
46 oyeye-*RR*

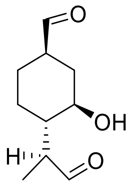
47 oyeye-*RS*

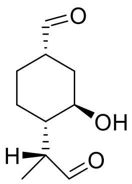
48 oyeye-*SR*

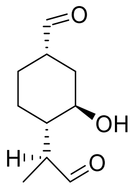
49 oyeye-*SS*

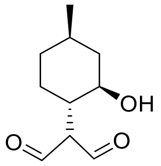
50 oeeyy

formula: $C_{10}H_{18}O$ (molar mass 154.25, total 1 compound)

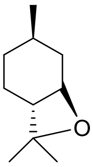
51 oeoee-4D

formula: $C_{10}H_{18}O_2$ (molar mass 170.25, total 1 compound)

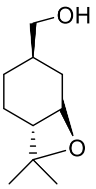
52 oooee-4D

formula: $C_{10}H_{16}O_2$ (molar mass 168.23, total 2 compounds)

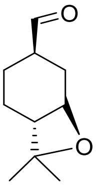
53 oyoee-4D-*R*

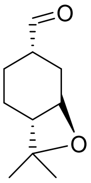
54 oyoee-4D-*S*

formula: $C_{10}H_{16}O_3$ (molar mass 184.23, total 2 compounds)

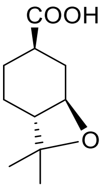
55 oxoee-4D-*R*

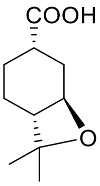
56 oxoee-4D-*S*

formula: $C_{10}H_{20}O_4S$ (molar mass 236.33, total 1 compound)

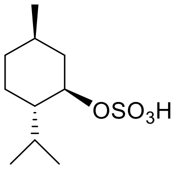
57 seeee

formula: $C_{10}H_{20}O_5S$ (molar mass 252.33, total 3 compounds)

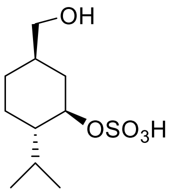
58 soeee

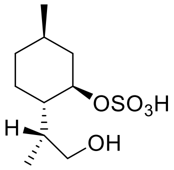
59 seeoe-*R*

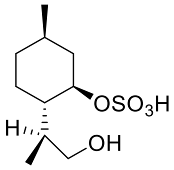
60 seeoe-*S*

formula: $C_{10}H_{20}O_6S$ (molar mass 268.33, total 2 compounds)

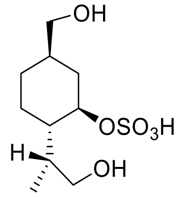

61 soeoe-*R*

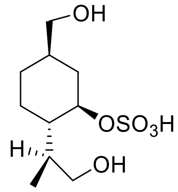

62 soeoe-*S*

formula: $C_{16}H_{28}O_7$ (molar mass 332.39, total 1 compound)

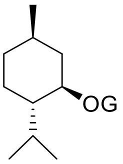

63 Oeeee

formula: $C_{16}H_{28}O_8$ (molar mass 348.39, total 8 compounds)

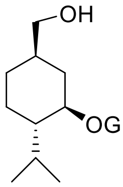

64 Ooeee

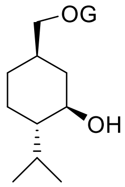

65 oOeee

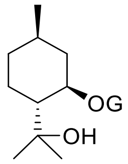

66 Oeoee

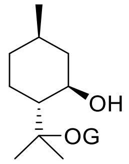

67 oeOee

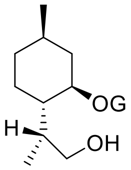

68 Oeeoe-*R*

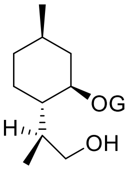

69 Oeeoe-*S*

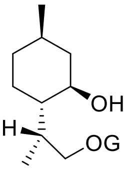

70 oeeOe-*R*

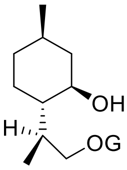

71 oeeOe-*S*

formula: $C_{16}H_{28}O_9$ (molar mass 364.39, total 6 compounds)

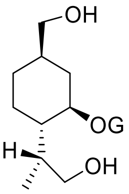

72 Ooeoe-*R*

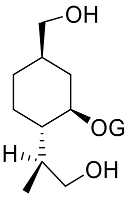

73 Ooeoe-*S*

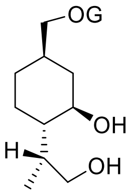

74 oOeoe-*R*

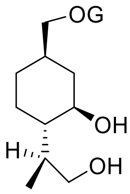

75 oOeoe-*S*

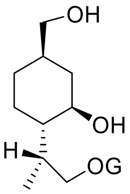

76 ooeOe-*R*

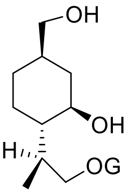

77 ooeOe-*S*

formula: $C_{16}H_{26}O_8$ (molar mass 346.37, total 4 compounds)

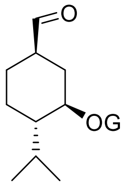

78 Oyeee-*R*

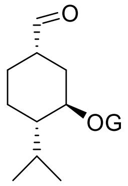

79 Oyeee-*S*

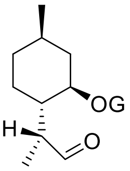

80 Oeeye-*R*

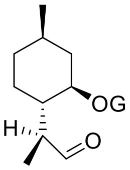

81 Oeeye-*S*

formula: $C_{16}H_{26}O_9$ (molar mass 362.37, total 16 compounds)

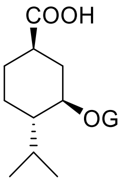

82 Oxeee-*R*

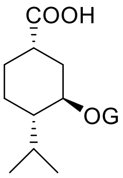

83 Oxeee-*S*

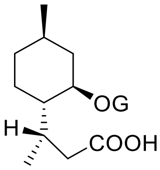

84 Oeexe-*R*

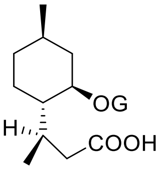

85 Oeexe-*S*

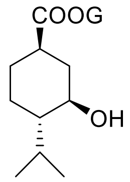

86 oXeee-*R*

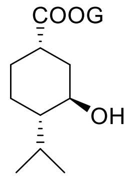

87 oXeee-*S*

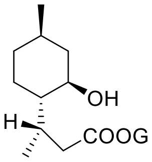

88 oeeXe-*R*

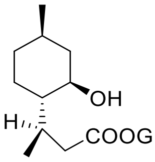

89 oeeXe-*S*

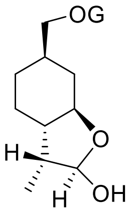

90 oOeye-5A-*RR*

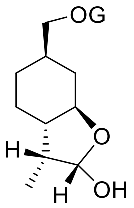

91 oOeye-5A-*RS*

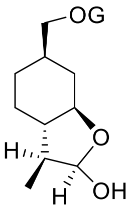

92 oOeye-5A-*SR*

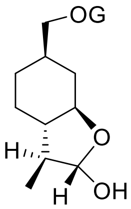

93 oOeye-5A-*SS*

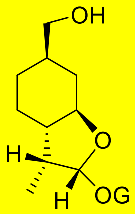

94 ooeYe-5A-*RR*

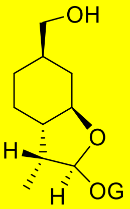

95 ooeYe-5A-*RS*

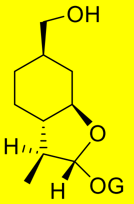

96 ooeYe-5A-*SR*

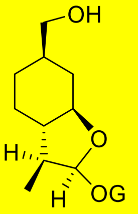

97 ooeYe-5A-*SS*

formula: $C_{16}H_{24}O_9$ (molar mass 360.36, total 5 compounds)

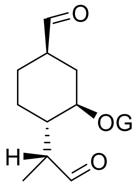

98 Oyeye-*RR*

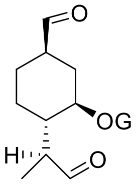

99 Oyeye-*RS*

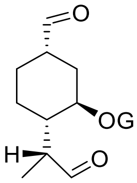

100 Oyeye-*SR*

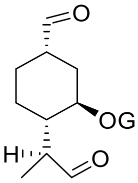

101 Oyeye-*SS*

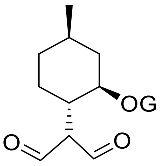

102 Oeeyy

Notes

- Compounds are highlighted in yellow when their *RS* designator changes from their parent compounds.
- G stands for a glucuronyl group:

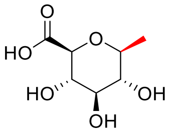

Supplement: S1 Table — (PDF) [file pone.0216577.s004.pdf]
